# Chemical and cultural control of *Tropilaelaps mercedesae* mites in honeybee (*Apis mellifera*) colonies in Northern Thailand

**DOI:** 10.1371/journal.pone.0188063

**Published:** 2017-11-10

**Authors:** Jeffery S. Pettis, Robyn Rose, Veeranan Chaimanee

**Affiliations:** 1 Bee Research Laboratory, USDA-ARS, Beltsville, Maryland, United States of America; 2 Plant Protection and Quarantine, USDA-APHIS, Riverdale, Maryland, United States of America; 3 Department of Biotechnology, Maejo University Phrae Campus, Rong Kwang, Phrae, Thailand; Nanjing Agricultural University, CHINA

## Abstract

At least two parasitic mites have moved from Asian species of honeybees to infest *Apis mellifera*. Of these two, *Varroa destructor* is more widespread globally while *Tropilaelaps mercedesae* has remained largely in Asia. *Tropilaelaps* mites are most problematic when *A*. *mellifera* is managed outside its native range in contact with Asian species of *Apis*. In areas where this occurs, beekeepers of *A*. *mellifera* treat aggressively for *Tropilaelaps* and *Varroa* is either outcompeted or is controlled as a result of the aggressive treatment regime used against *Tropilaelaps*. Many mite control products used worldwide may in fact control both mites but environmental conditions differ globally and thus a control product that works well in one area may be less or ineffective in other areas. This is especially true of volatile compounds. In the current research we tested several commercial products known to control *Varroa* and powdered sulfur for efficacy against *Tropilaelaps*. Additionally, we tested the cultural control method of making a hive division to reduce *Tropilaelaps* growth in both the parent and offspring colony. Making a split or nucleus colony significantly reduced mite population in both the parent and nucleus colony when compared to un-manipulated control colonies. The formic acid product, Mite-Away Quick Strips®, was the only commercial product that significantly reduced mite population 8 weeks after initiation of treatment without side effects. Sulfur also reduced mite populations but both sulfur and Hopguard® significantly impacted colony growth by reducing adult bee populations. Apivar® (amitraz) strips had no effect on mite or adult bee populations under the conditions tested.

## Introduction

Honeybee colonies are susceptible to a variety of pests and diseases and beekeepers may use cultural, genetic and chemical control to try and limit the impact of these threats. Some examples of each for controlling the mite *Varroa* are; (cultural) dividing a colony into a nucleus colony and the parent, (genetic) mite-resistant selected bee stocks and (chemical) the use of amitraz in a slow release strip. When chemical controls are used on parasitic mites the beekeeper is trying to control one arthropod on another (mite on an insect) and thus toxicity can be an issue for the host bees as well. Mite control products are known to impact colony health [1]. Specifically, miticides used to control *Varroa* mites accumulate in wax [2] and can impact drone [3], queen [4–6] and colony health [1,7]. Most research on control measures has been conducted with *Apis mellifera* (European honey bees) and *Varroa destructor*. Another mite parasite of bees, *Troplilaelaps* sp. [8] is found in Asia and has received less attention but this mite threatens beekeeping worldwide and adequate control measures are needed.

*Tropilaelaps* mites are very similar to *Varroa* as both are honeybee ectoparasites that feed on immature developing bees (brood). Mite parasitism can cause brood mortality and colony decline [9]. Currently four species of *Tropilaelaps* are recognized [10], *Tropilaelaps clareae* Delfinado and Baker, *T*. *mercedesae* Anderson and Morgan, *T*. *thaii* Anderson and Morgan and *T*. *koenigerium* Anderson and Morgan (henceforth collectively referred to as *Tropilaelaps*). *Tropilaelaps* mites have expanded their host range as *A*. *mellifera* were introduced into Asia [10]. The giant honeybee (*Apis dorsata* F.) is thought to be the original host of *Tropilaelaps* [10]. *Tropilaelaps* mites now pose a major threat to managed *A*. *mellifera* [10]. *Tropilaelaps* mites reproduce rapidly and have a shorter phoretic stage than *Varroa*; thus, they may outcompete *Varroa* when both mites are present [11–12]. Rapid reproduction and recent range expansion make *Tropilaelaps* an emerging threat to managed honeybees worldwide [9, 13] and proven control measures are needed.

To investigate possible cultural and chemical control for *Tropilaelaps* the following three experiments were conducted; 1&2) testing three commercially available *Varroa* control compounds and powdered sulfur for efficacy against *Tropilaelaps* in colonies in both the wet and dry season in Thailand; 3) testing the cultural control method of dividing *Tropilaelaps*-infested colonies (parent and nucleus colonies).

## Materials and methods

Honeybee colonies (*A*. *mellifera*) were managed at Maejo University Phrae Campus, Rong Kwang, Phrae Thailand in three apiaries all within a 1 km radius on campus. Colonies had received no mite treatments in the previous four months and all colonies were sampled for the level of *T*. *mercedesae* prior to mite control testing. Frames containing sealed worker brood were removed from colonies and brought into the lab and 100 brood cells were individually uncapped and using forceps and a small light the larvae/pupae were removed and brood and cells were inspected for the presence of *T*. *mercedesae* [9, 12–15]. Groups of 10 cells in a line were opened and the observer moved at random across the sealed brood area, most often opening 50 cells on two sides of a single brood frame. In 20 hives, a second 100 cells were opened when low or zero infested cells were found in order to gain insight into the sensitivity of 100 cells as an indicator of brood infestation rate.

### Chemical control experiments #1 (wet season) and #2 (dry season)

Colonies were assessed for adult bee and mite populations and then assigned to one of five (wet season) or four (dry season) treatment groups with 10 colonies per treatment utilized. Colonies were ranked from high to low mite infestation and treatments assigned down the rank (stratified random) in groups of five colonies (experiment #1) or groups of four (experiment #2) to ensure balanced mite levels across all treatment groups. Treatments consisted of untreated controls, formic acid, one pad of Mite-Away Quick Strips® applied twice at 0 and day 14, sulfur powder, 20 grams applied three times across the end bars at day 0, 7 & 14, Hopguard®, two strips applied twice at day 0 and 14 and amitraz, two Apivar® strips, applied once and left in place for 42 days. The second control experiment differed in treatments in that the amitraz treatment was eliminated, the sulfur treatment was reduced to 20 grams applied twice at day 0 and 14 and Hopguard® was reduced to a single strip applied on days 0 and 14. These reductions in dose or timing were based on observed negative effects on bee populations in experiment 1.

Colonies were monitored for adult bee population density and sealed brood levels at three time intervals, pre-treatment (day 0) and at ca. 30 and 60 days following the initiation of treatments. Adult bee population density consisted of a visual inspection of each comb and adult bee coverage estimated to the nearest 0.5 frame coverage (1 = fully covered in adult bees). Sealed brood was estimated using a 5x5cm square clear grid such that 32 squares would constitute one side of a Langstroth deep frame covered in sealed brood. The first control experiment began on 24 August 2015 and ended on 30 October 2015. The second experiment began on 4 December 2015 and ended on 6 February 2016. Fifty colonies were utilized in experiment #1 and 40 colonies in experiment #2 (n = 10 colonies/treatment).

The software SAS (SAS Institute Inc. 2015) and StatXact (Cytel Software Corp) were used for statistical analysis. The colony was the experimental unit. The variable Mites was modeled as 2-factor generalized mixed models using Proc Glimmix (SAS Institute) with Treatment and Day as the repeated factor. When this model could not provide results, a non-repeated model was used. When the outcome was zero for a treatment-day count then exact chi-square tests (StatXact, Cytel Software Corp) were also used to analysis the data. Frame was modeled as 2-factor mixed models using Proc Mixed with Day as the repeated factor and the sp(pow) variance-covariance structure. All pair-wise comparisons were done with Sidak adjusted significance levels to hold the experiment error-rate at 0.05.

### Cultural control experiment

Six colonies were randomly selected from a group of 65 mite-infested colonies for inclusion in the cultural control experiment. Six additional colonies (6 of 10 of the untreated control colonies) in experiment #1 were used as undisturbed controls for the cultural control experiment as they ran simultaneously in two apiaries within 200 meters of each other. The six colonies selected to be split were examined, the queen located and left in the parent colony, then two frames of brood and one frame of honey, all with adhering bees were transferred into a new closed hive body. One additional frame covered in adult bees was shaken into the hive and mated queens from the same local stock were added in queen cages with a candy slow release mechanism and the colonies transported to a new location and remained closed for two days to reduce bee fly-back to parent colonies. Colonies were fed sucrose syrup during closure to aide in queen acceptance. Colonies were inspected on day 7 following relocation and one new mated queen was added to one colony that had rejected the first queen, in all other colonies the queens had been accepted. Mite levels were assessed, as described above, pretreatment (0 day), and 15 days, 1.5 and 2.5 months following nucleus removal from parent colonies. Frames covered in adult bees were also determined, as described above, to the nearest 0.5 frame on day 0 and 1.5 and 2.5 months.

In the nuclei experiment the frames of bees and mite-infested cells were analyzed as a repeated ANOVA model SAS (SAS Institute Inc. 2015). All pair-wise comparisons were done with Sidak adjusted significance levels to hold the experiment error-rate at 0.05.

## Results

### Chemical control experiment #1 wet season

**Fig 1:**
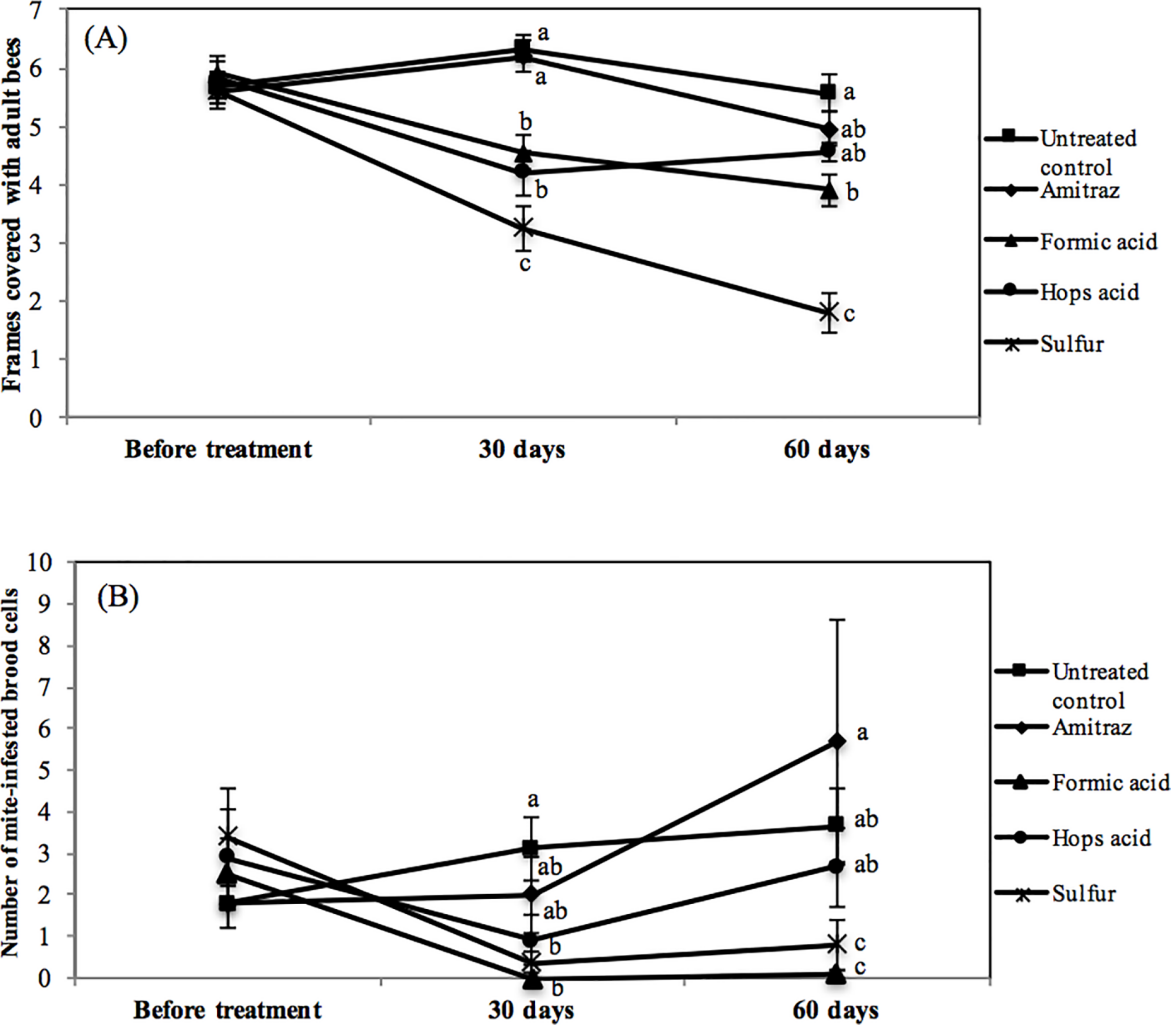
**Average (mean ± SEM) number of frames covered in adult bees (A) and number of *Tropilaelaps*-infested cells observed by uncapping 100 sealed brood cells (B) from honey bee colonies treated with four different mite control products and untreated controls in the wet season. Treatment period was ca. 30–45 days dependent upon control product.** Letters indicate significant differences between treatment means (ANOVA, P < 0.05).

The fifty colonies utilized did not differ in starting adult bee population when assigned to the five treatment groups, averaging just below six frames of bees (Fig 1A). Following the treatments at day 30, hops and formic acid along with sulfur all had significant negative effects on adult bee population (F = 20.91, 4 df, p = 0.0001; Fig 1A). By day 60 only the formic and sulfur treated hives had significantly fewer bees than controls, with sulfur colonies having only 1.8 frames of bees while controls had 5.5 (p < 0.05). Hops acid had a negative effect on adult population on day 30 but by day 60 the colonies had rebounded and were not different in strength from controls. Amitraz had no significant negative effect on adult bee population.

Mite populations were equal across treatments at the start of the experiment (Fig 1B) but 30 days following treatment mites had been reduced with the use of sulfur and formic acid (F = 2.0, 4df, p = 0.09) and by day 60 the mite levels in the formic acid and sulfur treated colonies were significantly lower than the controls (F = 3.29, 4df, p = 0.002). Hops acids and sulfur demonstrated some level of control while mite levels were unaffected by amitraz treatment.

### Chemical control experiment #2 dry season

**Fig 2:**
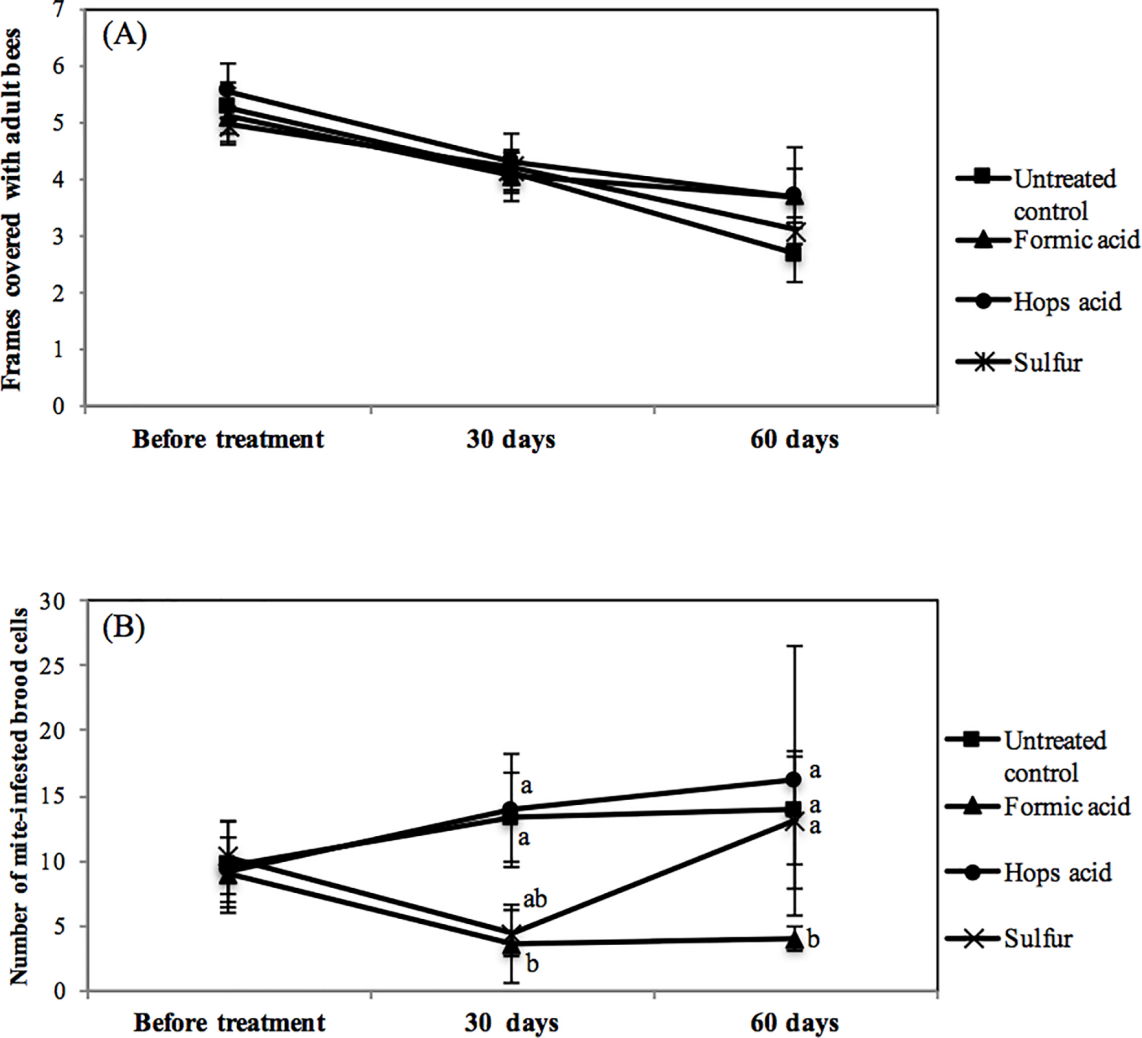
**Average (mean ± SEM) number of frames covered in adult bees (A) and number of *Tropilaelaps*-infested cells observed by uncapping 100 sealed brood cells (B) from honey bee colonies treated with three different mite control products and untreated controls in the dry season. Treatment period was ca. 30–45 days dependent upon control material.** Letters indicate significant differences between treatment means (ANOVA, P < 0.05).

The forty colonies utilized did not differ in adult bee population when assigned to the four treatment groups, averaging just over five frames of bees (Fig 2A). Following the treatments at day 30 and 60 all colonies had lost adult bee populations but no treatment effects were noted (F = 0.10, 3df, p = 0.95, Fig 2A).

Mite populations were equal across treatments at the start of the experiment (Fig 2B). At both 30 and 60 days following formic treatment, mites had been significantly reduced with the use of formic acid (F = 4.51, 3df, p = 0.008). Mite levels were lower in sulfur treated hives at day 30, but not significantly different from controls. Hops acids did not affect mite levels.

### Cultural control nuclei experiment

**Fig 3:**
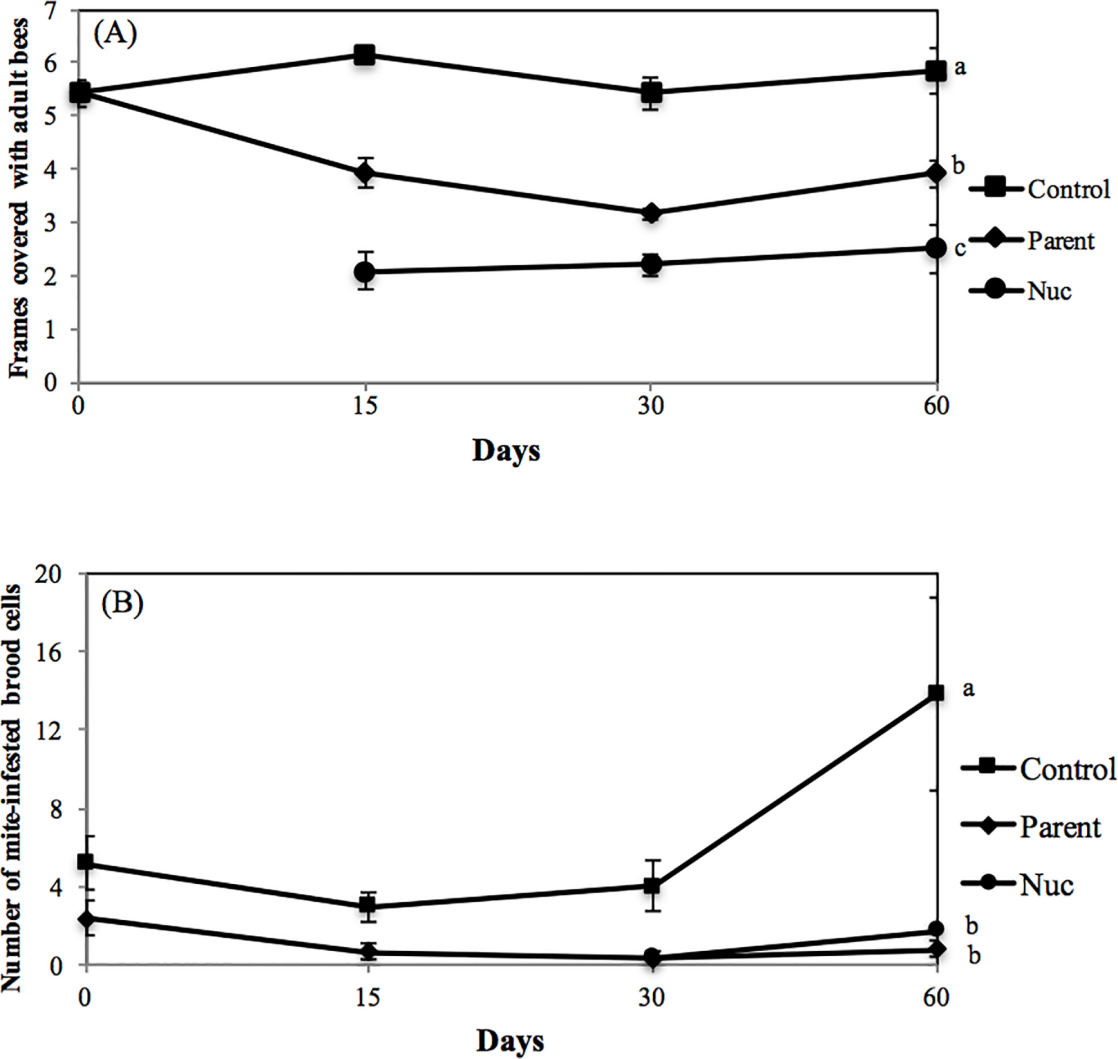
**Average (mean ± SEM) number of frames covered in adult bees (A) and number of *Tropilaelaps*-infested cells observed by uncapping 100 sealed brood cells (B) from parent honey bee colonies that were used to make a single nucleus colony or unmanipulated control colonies.** Letters indicate significant differences between treatment means (ANOVA, P < 0.05).

Colonies that served as controls were of similar size to the colonies (parents) used to make nuclei (5.41 ± 0.15 and 5.41 ± 0.23, mean ± SEM) and were not significantly different (see Fig 3A). The removal of bees and brood to make the nucleus colonies reduced the overall colony population when compared to non-manipulated controls (day 15, Fig 3A). At the end of the experiment the nuclei and parent colonies were significantly smaller than the controls (Fig 3A; 2.2 and 3.8 vs. 5.8 frames of bees, F = 68.6, 2 df, p < 0.0001). Sixty days following the colony divisions, mite populations in both the parent and nuclei colonies were significantly lower than controls (Fig 3B; F = 18.2, 2df, p < 0.0001).

## Discussion

Chemical control of *Tropilaelaps* was demonstrated with the use of formic acid and even the local mite control powdered sulfur, achieved a significant level of mite control. Both substances negatively impacted adult bee population and the beekeepers in Thailand use sulfur at specific intervals and when nectar flows allow the colonies to recover. Formic acid was the only commercial product tested that significantly reduced mite levels and the slight negative impact on colony growth is perhaps a fair tradeoff to achieve mite control. The cultural control of making a colony division significantly reduced mite populations in both the parent and nucleus over a 60 day period. This is an encouraging finding and supported by previous research on *Varroa* that shows similar impact on *Varroa* growth by making colony divisions [16].

The *Varroa* control products tested did not all work on *Tropilaelaps*. Most surprising was the ineffective nature of amitraz (Apivar®). It works as a contact on *Varroa* and is dependent on bees to move about the colony and contact *Varroa* and it simply did not work on *Tropilaelaps* under the colony conditions tested in Thailand. This may point to the differences in phoretic behavior between the two parasites. *Varroa* spends a phoretic phase on adult bees once new females emerge from brood cells [17] while *Tropilaelaps* do not require this time on adult bees but instead emerge from cells and move about on the comb to locate new brood cells to infest [18]. Because of the behavior of mites to remain on the comb and or in brood cells, even if adult bees have sufficient amitraz circulating on their body, it may not be sufficient to allow for mite control. We propose that the lack of phoresy on adult bees is the reason amitraz did not work on *Tropilaelaps* but works well on *Varroa*. Both fluvalinate and flumethrin are sold in a slow release form for mite control in Thailand and one must assume they work to some degree on *Tropilaelaps*. Thus it is surprising that we found no efficacy of amitraz in a slow release strip. Hopguard® did not show efficacy in these trials and again it suggest that the amount circulating on the body of adult bees is insufficient to control *Tropilaelaps*. The higher dose of Hopguard® tested in experiment #1 nor half that dose in experiment #2 gave adequate mite control. The lower dose Hopguard® used in experiment #2 did not adversely affect adult bee population as had the higher dose in experiment #1.

Powdered sulfur did significantly reduce mite populations but the dose and timing of application appear to be critical to limiting negative impact on adult bees and brood. Local beekeepers have used powdered sulfur for many years as a means to control *Tropilaelaps* but discussions with them indicated that the dose and the time of year can be critical to not harm the colony. Dust such as sulfur are known to be toxic to uncapped brood [19] and thus it is not surprising that brood and adult bee populations were impacted in experiment #1 with sulfur applied each week for three weeks. In Experiment #2 the total dose was reduced and less impact was observed on adult bee populations but there was also less mite control. Mites levels were reduced on day 30 but by day 60 the mite populations had rebounded and were similar to untreated controls (Fig 2B and S1 File). Formic acid use resulted in the highest level of mite control across both experiments with some adverse effects on adult bee population in experiment #1. Formic acid is a volatile product and as such can reach mites on the comb as well as perhaps mites under the capped cells as has been demonstrated with formic acid use and *Varroa*. The formic acid product tested has a slow release mechanism and should give control over an extended period of time to allow for control of mite emerging from brood cells. Earlier research [20] had demonstrated that formic acid could control *Tropilaelaps* so it is not surprising that in the current studies formic acid was an efficient control for this mite. However, formic remains problematic in the risk it poses to the applicator and it can be corrosive on equipment. To our knowledge formic acid is not in widespread use in Thailand to control *Tropilaelaps* despite the encouraging results of these studies. Perhaps improvements in education of beekeepers in the proper use of formic products might increase it safe use.

Cultural control options are needed to allow for a more integrated approach to parasitic mite control and reduce the use of chemicals within the hive and the concurrent risk of residues in wax and honey. The current results indicate that the removal of frames of bees and brood to start a new colony (nucleus) results in lower mite levels in the new and parent colony from which the bees were removed. This is similar to results with *Varroa* where the same effects have been demonstrated [16]. The current findings should be further tested with higher colony numbers and in different locations in Asia to be sure the current findings are robust and applicable to other areas where *Tropilaelaps* occur. However, the results to date indicate that making a nucleus colony can significantly reduce the mite population in both the nucleus and the parent colony.

## Supporting information

S1 File Contains all supporting information. (XLSX)
